# Evaluating the responses of a territorial solitary carnivore to potential mates and competitors

**DOI:** 10.1038/srep27257

**Published:** 2016-06-02

**Authors:** Maximilian L. Allen, Veronica Yovovich, Christopher C. Wilmers

**Affiliations:** 1 Center for Integrated Spatial Research, Environmental Studies Department, University of California, Santa Cruz, USA; 2 Department of Forest and Wildlife Ecology, University of Wisconsin, 1630 Linden Drive, Madison, WI 53704, USA.

## Abstract

Successful communication is critical to the fitness of individuals and maintenance of populations, but less is known regarding the social contexts and reactions to scent marking by other individuals in solitary carnivores, including pumas. We evaluated the responses of resident male pumas to visitation and scent marking by potential competitors (other male pumas) and potential mates (female pumas) by capturing and marking 46 pumas (*Puma concolor*), and documenting scent marking behaviours using motion-triggered video cameras. By comparing resident male puma visitation rates and communication behaviours in response to either male or female visitors, we found that their visitation and communication behaviours were best explained by the combination of visitation by both competitors and potential mates. Resident males returned to scent marking sites more quickly and increased their rate of flehmen response after visitation by a females, while they increased their rate of visitation and duration of visits in response to other males. Male pumas also visited less frequently in summer and autumn when female visitation rates were lower, but males created nearly twice as many scrapes during these visits. This study suggests that advertising for mates when scent marking may sometimes overshadow the importance of deterring competitors and claiming territory.

Communication is an integral element of animal behaviour, and scent marking is one of the most frequent forms of communication for many mammal species. As secondary sexual characteristics, scent marks are considered reliable indicators of an individual’s health and viability as a mate^1^,^2^. Due to their spatially dispersed populations, solitary carnivores rely on scent marking as an indirect method of conspecific communication (e.g. ref. 3, 4, 5, 6). The predominance of communication via scent marking has led to many explanations regarding the function of this behaviour in solitary carnivores. The main functions are thought to include asserting dominance^7^,^8^, selecting mates^4^,^9^,^10^, and maintaining territories^3^,^11^,^12^,^13^. Scent marking may also be used to determine the identity and relatedness of individuals^14^, to mark and defend food resources^15^,^16^, and to possibly avoid infanticide^6^. Given the many different purposes attributed to scent marking, understanding the dynamics influencing this behaviour will help us understand its use in social contexts.

The two most important functions of scent marking in solitary carnivores may be to advertise their reproductive status to potential mates and to assert dominance and maintain territories over their competitors. For solitary carnivore species with a defined breeding season, scent marking increases during the breeding season^4^,^5^,^11^,^17^, which suggests that scent marking plays an important role in advertising for potential mates. However, scent marking also occurs throughout the year^3^,^4^,^6^,^12^,^18^, suggesting it has an on-going role in maintaining territories, and may therefore play an essential role in social organization stability. Many communication systems, including among solitary carnivores, are mark-countermark systems, whereby one individual marks, and another then marks in response^3^,^12^. The infrequency of encounters among solitary carnivores makes it more likely for individuals to respond when they come across intraspecific communication attempts, whether through investigation, scent marking, or another response. The utilities of scent marking in solitary carnivores could be further understood by determining how they respond to competitors and potential mates.

In contrast to the majority of carnivore species, many large felids breed throughout the year^17^,^18^. This makes them ideal species to discern the relative importance between scent marking purposes, including whether individuals react more strongly to visitation and scent marking by potential mates or by potential competitors. Like other solitary felids, pumas are territorial^18^,^19^, and communicate most frequently through scent marking^18^,^20^,^21^. Male pumas actively compete for territories that encompass resources, including access to potential mates, and scent mark throughout their territories^19^. Some puma populations, including in our area, show a birth pulse in summer^22^, with a related peak in mating season in late winter or spring.

The most frequent form of scent marking by pumas is scraping^18^,^20^,^21^ (Supplementary Video 1), which is concentrated in areas called ‘community scrapes’ (e.g. ref. 20) or ‘shared scrapes’ (e.g. ref. 18). We use the term ‘community scrapes’ hereafter. These are defined as a location in which multiple pumas create scrapes in a small area (>3 scrapes in 9 m^2^) that is not associated with a kill or resting site^20^. Male visitation and scent marking at community scrapes occurs regularly throughout the year^10^,^18^. In contrast, female visitation occurs in short bouts of activity that can be at any time of the year, which most likely correlate with breeding and estrus^10^,^18^. Pumas also exhibit other communication behaviours at community scrapes such as olfactory investigation (Supplementary Video 2), the flehmen response (Supplementary Video 3), body rubbing (Supplementary Video 4), and caterwauling or other vocalizations (e.g. ref. 10,20,21,23). Little research has been conducted as to which stimuli elicit communication behaviours, or how these behaviours and their frequency are affected by interactions with potential mates and competitors.

We monitored a puma population with 46 marked individuals from 2011–2014 in order to better understand how male pumas respond to visitation and scent marking by male competitors and potential female mates. We deployed motion-triggered video cameras at community scrapes to record communication behaviours. Our specific objectives were to: 1) determine whether seasonal variation occurred in visitation and scent marking of male pumas at community scrapes. We hypothesized that if visitation and scent marking were based on competitors they would not vary throughout the year, while if they were based on potential mates than visitation rates would be most frequent and the most scrapes would be created in spring to coincide with the perceived peak in the mating season. 2) Determine how male visitation and behaviours changed in response to visitation by possible mates and competitors. We hypothesized that male pumas would visit and exhibit behaviours more frequently after a recent visit by both potential mates and competitors. Additionally, we hypothesized that if potential mates drive male communication, their visitation and display of behaviours would increase in response to visitation by potential mates rather than to visitation by competitors. 3) Determine whether male pumas display stronger responses to visitation by potential mates, competitors, or whether it is a combination of both. We hypothesized that while visitation and scent marking is likely affected by the combination of both, the function of visitation and scent marking may be more affected by potential mates, as they may be the rarest resource in a male’s territory.

## Results

We monitored 28 community scrape areas for a mean of 596 (±45 SE) days. We recorded 724 visits by mature males, and 198 visits by mature females traveling without cubs. We used the 533 visits by the 11 known (collared) mature males in our analyses.

### Seasonal Variation in Visitation

**Figure 1:**
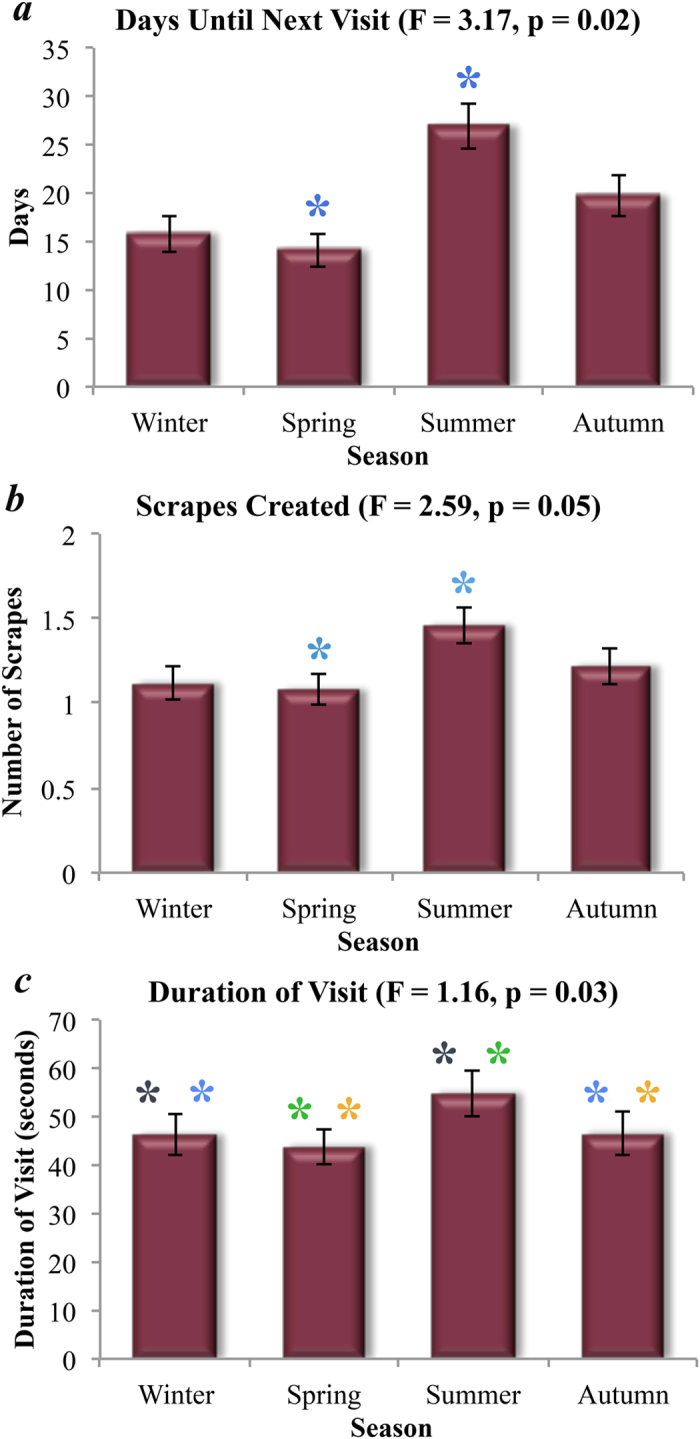
The seasonal means for 3 aspects of male puma behaviour: days until next visit (*a*), number of scrapes created (*b*), and duration of visit (*c*). For each behaviour we report the mean, the standard error represented as error bars, as well as the F score and p-values from our ANOVA, and significant differences from our post hoc Tukey HSD tests with asterisks of the same colour.

Male visitation behaviours varied among seasons; including the days until next visit (*F*_*3, 445*_ = 3.17, *p* = 0.02), the number of scrapes created (*F*_*3, 519*_ = 5.60, *p* < 0.01), and the duration of visits (*F*_*3, 519*_ = 3.00, *p* = 0.03). The days until next visit for males in spring was nearly 2 times more frequent than in summer (p = 0.01) (Fig. 1a). In summer, males created 35% more scrapes than in spring (p = 0.02), and 30% more than in winter (p = 0.01). In autumn, males created 13% more scrapes than in spring (p = 0.04) and 9% more than in winter (p = 0.01) (Fig. 1b). The duration of male visits in summer was 25% longer than in spring (p = 0.05), and marginally longer than in winter (19%, p = 0.08) (Fig. 1c).

**Figure 2:**
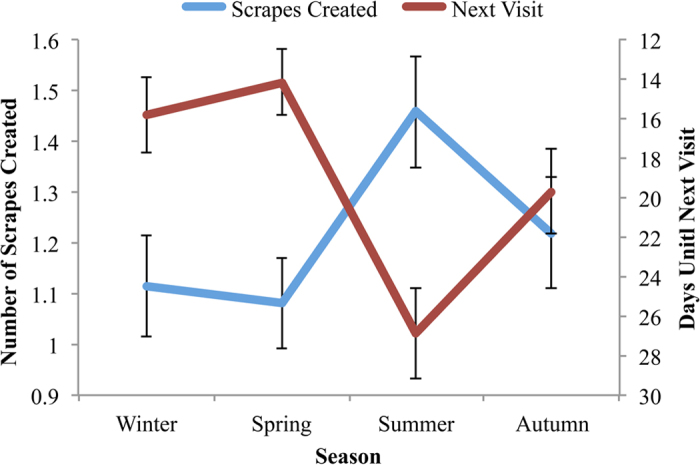
The days until next visit and scrapes created by male pumas during each season. Each variable is represented as its mean value for the season, with error bars representing the standard error. The values have a significant inverse relationship, suggesting that male pumas create more scrapes in seasons with less frequent visits.

A post hoc test showed that the seasonal mean for days until next visit had strong inverse correlations with both the number of scrapes made during visits (r^2^_1, 3_ = 0.94, p < 0.01), and visit duration (r^2^_1, 3_ = 0.93, p = 0.04). The inverse relationship between the number of scrapes created and days between visits suggests that male pumas create more scrapes during seasons with less frequent visitation (Fig. 2). The inverse relationship between the visit duration and days between visits suggests that males spend longer durations at the scrape during seasons with less frequent visitation.

### Influences on male visitation and behaviours

Male days until next visit and visit duration increased in response to visitation by other pumas, while their number of scrapes created did note vary significantly (Table 1). The mean time between visits for male pumas was 37% more frequent when another male was present within 28 days (*F*_*1, 447*_ = 12.09, *p* < 0.01), and 34% more frequent when another male was present within 7 days (*F*_*1, 447*_ = 7.34, *p* = 0.01). The mean time between visits for males was 29% more frequent when a female was present within 28 days (*F*_*1, 447*_ = 18.80, *p* < 0.01), and also 29% more frequent when a female was present within 7 days (*F*_*1, 447*_ = 15.68, *p* < 0.01). Duration of visit was significantly influenced by visits within 7 days by other males, increasing by 31% when another male was present (*F*_*1, 455*_ = 4.33, *p* = 0.04), but females did not have a significant effect (Table 1). The responses of male pumas to visitation by competitors and potential mates suggest that both are important driving factors in their visitation.

For the display of behaviours, the display of flehmen response and body rubbing increased significantly in response to visitation by other pumas, while scraping and olfactory investigation did not (Table 2). Flehmen response increased in response to female visitation in both time periods, increasing by 9.4% when a female was present in the previous 28 days (*X*^2^_1, 463_ = 11.37, *p* < 0.01, *phi* = 0.17), and by 13.1% when a female was present in the previous 7 days (*X*^2^_1, 463_ = 14.74, *p* < 0.01, *phi* = 0.19). Flehmen response also increased 8.3% in response to male visitation in the previous 7 days (*X*^2^_1, 463_ = 5.29, *p* = 0.02, *phi* = 0.12). Body rubbing increased 9.8% in response to male visitation in the previous 28 days (*X*^2^_1, 463_ = 10.52, *p* < 0.01, *phi* = 0.16), but was not significantly influenced by visits by females (Table 2).

### Comparative effects of competitors and potential mates

We compared the potential influences on days until next visit, the visit duration, body rubbing and the display of flehmen response by male pumas. Among the five models we tested, the model combining female and male visitation was the clearly the top model for all the variables we tested; all other models were implausible (Table 3). These top models supported our hypothesis that both competitors and potential mates were important drivers of male puma behaviour.

## Discussion

Due to their spatially dispersed populations, scent marking is an important mechanism for communicating with potential mates and competitors among solitary carnivores. We analysed the use of scent marking behaviours at community scrapes by male pumas to determine whether they exhibited stronger reactions through visitation or communication behaviours to either potential mates or by competitors. Our results support previous theory that has suggested that scent marking is multi-purpose and used for both competitors and potential mates. Based on visitation to community scrapes, we found that potential mates sometimes created a stronger response than competitors for male pumas. The best model explaining male puma visitation and scent marking behaviours, however, was the combination of potential mates and competitors. We also found seasonal variation in both visitation and the number of scrapes created by male pumas, and this may be due to the seasonal visitation patterns of female pumas.

The combination of potential mates and competitors was clearly the best explanatory model for each variable we tested. Solitary carnivores rely upon scent marking for intraspecific communication; including scent marking to advertise for potential mates^4^,^9^,^10^, and demonstrating the use of an area to maintain territories from competitors^3^,^11^,^12^,^13^,^18^. Our results suggest that scent marking is used for multiple purposes, but our results suggest there may be differences in how male pumas react to visits by potential mates and competitors. Future research to determine how potential mates and competitors affect the functional use of scent marking could increase our understanding of behavioural ecology.

Female pumas are less frequent visitors to community scrapes^10^, and we found that when females visit, male pumas show a larger increase in their visitation rate than they do for male puma visits. Male solitary carnivores often compete for territories with food, mates, and other resources, but spatial and temporal unpredictability may make potential mates the most limited resource for which males compete (e.g. ref. 24). Female pumas can be in estrus at any time of year, and likely visit community scrapes at these times in search of breeding opportunities^10^,^18^. Female pumas often select the males that visit most frequently to breed with^10^, making it important for a male to be the dominant resident in order to maximize his exposure to community scrapes when a receptive female is present. Scent marking to define a territory may be helpful for many resources, but complete exclusion is seldom achieved^10^. Since the currency for winning the ultimate evolutionary game is successfully producing offspring, securing potential mates could be a more important reason for holding a territory than food or other resources.

Contrary to our hypothesis, the number of scrapes created during a visit was apparently not a direct response to either potential mates or competitors. Instead, the number of scrapes created was inversely correlated with visitation, as male pumas created more scrapes during seasons when they visited less frequently. Scent marking is used in some species as a secondary sexual signal to advertise an individual’s health and viability as a mate^1^,^2^. In pumas, frequent visitation and scent marking, as a sign of dominance and territory ownership, is an important aspect of mate selection^10^. The increased number of scrapes could be a dishonest signalling behaviour (e.g. ref. 25) to over-represent their visitation and compensate for less frequent visitation during these seasons. By creating more scrapes during seasons with less frequent visitation, male pumas may be able to over-represent their actual visitation for mates, and therefore increase their chances of reproductive success. The greater number of scrapes were also created during the seasons with the hottest seasons, and male pumas may also be leaving more scent to compensate for it drying out. However, this does not explain why visitation is less frequent during these seasons. The seasonal changes in visitation may impact other aspects of puma ecology. For example, many male mammals lose weight during the breeding season because they prioritize securing mating opportunities over acquiring food^26^,^27^. Once the breeding season has past, individuals increase their foraging rate to recoup lost energy stores. We see similar shifts in puma foraging ecology where pumas in California increase their kill rates in summer and autumn^28^. If the same pattern applies to pumas, they may compensate for periods with lower energy acquisition by spending summer and autumn hunting more frequently, and visiting scrapes less frequently as an energy-saving measure.

In a social context it may be important to distinguish between the functional purposes of scent marking from the proximate frequency of scent marking. A key aspect of scent marking is for defining territories^3^,^11^,^12^, but the frequency with which a male must scent mark for maintaining boundaries is yet unknown. Scrapes are a combination of two forms of scent marking, with a physical scrape to help other pumas locate the scent, and urine to convey their messages^20^. Urine is also composed of multiple elements, including pheromones, and it is unknown how frequently the separate components need to be refreshed in order to attract mates. This may be a factor that necessitates the frequent visitation and scraping to community scrapes, with increases in visitation in the seasons when females are most likely to be present.

Visitation and the behaviours displayed by males were influenced by visitation of both females (visitation rate and flehmen response) and other males (visitation rate, duration of visits, flehmen response, and body rubbing). The strongest response, as measured by effect size, was in how quickly a male returned after a female visited (*d* = 0.49). Male pumas also increased their display of flehmen response after visitation by potential mates and competitors, which likely enables the male to investigate fresh scent more thoroughly. Males only exhibited more frequent body rubbing when another male was present within 28 days. It may be that body rubbing deposits scent in either larger quantities or in a way that persists longer in the environment. It may be effective in exhibiting their long-term presence to other males for territorial purposes, but may have limited utility to advertise for mate selection by females due to a lack of pheromones (e.g. ref. 29). Alternatively, body rubbing may disrupt or dampen the signals left by other males.

## Methods

### Study Area

**Figure 3:**
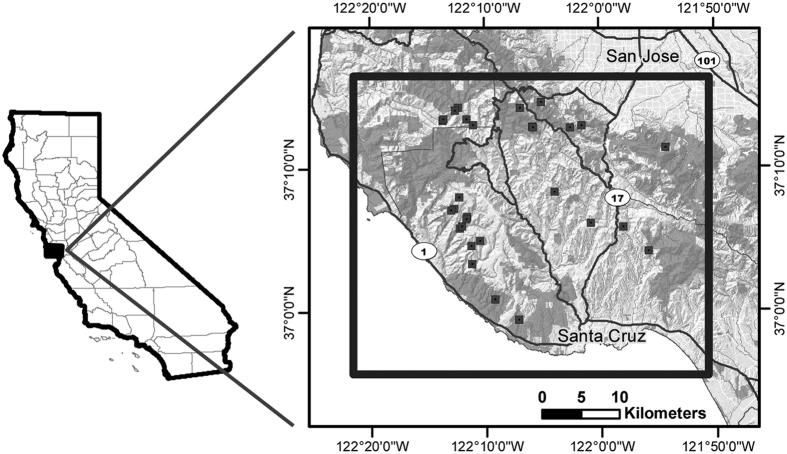
A map of the study area, created using ArcGIS (v. 10.1, ESRI, 2012, http://www.esri.com), which included areas in Santa Cruz, San Mateo, and Santa Clara Counties in California. The study area is outlined by the thick black line, within the greater context of major highways, and the cities of Santa Cruz and San Jose, and the location of each community scrape area monitored is noted.

Our study area encompassed 1,700 km^2^ in the Santa Cruz Mountains of California, including parts of Santa Cruz, San Mateo, and Santa Clara counties (Fig. 3). The boundaries of the study area included the Pacific Ocean to the west, Highway 84 and the city of San Jose to the north, the city of Santa Cruz to the south, and Highway 101 to the east. The highways and cities, including California Highway 17 that bisected the study area, were a major source of mortality for pumas in our study^30^, and also acted as barriers to movement; hence, these sites often formed the edges of puma home ranges. Vegetation characteristics and climatic conditions in the study area are classified as Mediterranean and have been described in detail elsewhere^30^.

### Field Methods

We monitored puma behaviour at community scrapes between May 2011 and January 2014 using motion-triggered video cameras with infrared flash (Bushnell TrophyCam model and TrophyCam HD model, Overland Park, KS). We initially found community scrapes by searching prominent landscape features and areas commonly used by pumas. We then developed a custom algorithm based on our algorithm for identifying kill sites with GPS data^30^. The algorithms identified potential community scrapes by locating clusters of >3 GPS locations where the locations were recorded more than 7 days apart from each other^30^. We documented 299 community scrapes during field investigations, and placed cameras at active community scrape sites. We programmed cameras to record a 60 s video for each time the camera was triggered, with a 1 s refractory period. To ensure spatially independent samples and also to avoid pseudo-replication, we pooled the data from cameras placed at community scrapes <1 km apart, except in cases where two scrapes were separated by a substantial travel barrier (e.g. an impassable ravine, river, etc.). We excluded any periods with camera malfunctions from our visitation samples.

Concurrent with camera deployment, we captured and placed GPS-enabled collars with unique visual identifiers on 46 pumas, and the capture methods we used are described in detail elsewhere^30^. All animal capture and handling, and experimental procedures were carried out in accordance with approved guidelines from the Independent Animal Care and Use Committee at the University of California, Santa Cruz (Protocols Wilmc0709 and Wilmc1101). When possible, we identified the individual pumas recorded in videos using unique collar identifiers, and subsequently placed the pumas into age and sex classes as: mature male, mature female, immature male, or immature female. For individuals without collars we determined the sex from visible characteristics including the position of the genitals. We removed immature pumas and mature females traveling with kittens from our analyses, as they tend to act as non-participants in mating behaviours^20^.

We quantified aspects of each video that met our qualifications, including the date and time, duration of visit to the closest second (averaged for pooled samples), and number of scrapes created (averaged for pooled samples). We also documented whether behaviours associated with intraspecific communication were displayed, including: scraping (where the puma clawed in substrate with their hind feet and then sometimes urinated and/or defecated on the scraped mound of material; Supplementary Video 1), olfactory investigation (where the puma used its olfactory sense to investigate cues and signals, as noted by the pumas nose within one head length of a scrape or other cue; Supplementary Video 2), flehmen response (where the puma picked up its head and curled back its upper lip, sometimes arching its neck backwards, in order to expose its vomeronasal organ; Supplementary Video 3), and body rubbing (where the puma rubbed its cheek or shoulder on the ground or an object, or rolled back and forth on the ground; Supplementary Video 4).

### Statistical Analyses

We used program *R* version 3.1.3^31^ for all statistical analyses, and following *R* guidelines we cited any associated packages used in analyses. In each analysis, we considered *p* ≤ 0.05 significant.

We wanted to determine whether seasonal variation occurred in visitation and scent marking of male pumas at community scrapes. We used mixed model analyses of variance (ANOVAs) to test for variation among seasons for 3 behaviours: ‘days until next visit’, ‘number of scrapes created per visit’, and the ‘duration of visit’. We first tested each data set for normality with a Shapiro-Wilk test and for variance equality with a Levene’s test^32^. Each data set lacked normality, and we therefore log transformed the data to meet the assumptions of the ANOVA, and then performed the ANOVA using the *nlme* package^33^. We used each of the behaviour variables as our dependent variables in the models, used season (based on the calendar year) as our fixed predictor variable, and included puma identity as a random effect to account for variation among individuals. When we found significant differences we used a post hoc Tukey’s HSD test^32^ using the *multcomp* package^34^ to determine where the significant differences occurred. We then performed post hoc analyses to determine whether the ‘days until next visit’ by male pumas was correlated with either the ‘number of scrapes created per visit’ or the ‘duration of visit’ among different seasons. In each analysis we used the seasonal means of each variable to perform linear regressions.

Next, we wanted to determine how male visitation and behaviours were influenced by the visitation of possible mates and competitors. First, we determined the influence of visitation by potential mates (females) and competitors (other males) for three aspects of visitation for male pumas: ‘days until next visit’, ‘number of scrapes created per visit’, and the ‘duration of visit’. We modelled each of the variables using a mixed model ANOVA, using the *nlme* package^33^. We used the behaviour variables as our dependent variables in the models, used a categorical predictor of presence or absence of other pumas (first other males, then females) for two time periods: one month (28 days) and one week (7 days) as our fixed predictor variable, and included the individual puma as random effects. We then calculated post hoc effect sizes using Cohen’s *d* score, and considered scores of 0.20 small effects, 0.50 medium effects, and 0.80 large effects^35^. Second, we determined the influence of recent visitation by potential mates and competitors on the display of four communication behaviours of male pumas: ‘scraping’, ‘olfactory investigation’, ‘flehmen response’, and ‘body rubbing’. We compared the proportion of visits each behaviour was displayed based on the presence of other pumas (first other males, then females) in the previous month using a chi-square test^32^. We then calculated post hoc effect sizes for each behaviour by calculating *phi* coefficients, using the *vcd* package^36^. We considered scores of 0.10 small effects, 0.30 medium effects, and 0.50 large effects^35^.

Last, we used model selection to determine whether male pumas display stronger responses to the visitation of a) potential mates, b) competitors, or c) a combination of both. We considered six models that included various permutations of male and female activity and season (Table 3). We used these models for each of the variables that we found to have a significant relationship in at least one time period (days until next visit, duration of visit, display of body rubbing, display of flehmen response). For our two continuous data sets (days until next visits and the duration of visit) we used a mixed model linear regression, after first log transforming the data to meet the assumptions of the test. For our 2 binomial data sets (display of body rubbing and flehmen response) we used a Generalized Linear Model with a binomial distribution and a logit link. We used the visitation and behaviour variables as our dependent variables in the models, the number of days since the last visit by male and female pumas as our fixed predictor variable, and the identity of the individual male puma as a random effect. Because the behaviours of male pumas are likely to be only influenced by relatively recent visits, we set our cut off for visits by other pumas at 50 days, roughly 3 times the mean 17.7 days between visits for male pumas^10^.

## Additional Information

**How to cite this article**: Allen, M. L. *et al.* Evaluating the responses of a territorial solitary carnivore to potential mates and competitors. *Sci. Rep.* **6**, 27257; doi: 10.1038/srep27257 (2016).

## Supplementary Material

Supplementary Video Legends

Supplementary Video 1

Supplementary Video 2

Supplementary Video 3

Supplementary Video 4

## Figures and Tables

**Table 1:** The influences of visitation by male and female pumas on visitation behaviours of male pumas.

Variable	Sex	Days	Mean (±SE)		*F*	*p*	*d*
			Present	Not Present			
Days Until Next Visit	Male	28	13.1 (2.1)	20.8 (1.3)	12.09	**<0.01**	0.32
		7	12.8 (2.5)	19.4 (1.3)	7.34	**0.01**	0.32
	Female	28	14.0 (2.2)	19.8 (1.2)	18.80	**<0.01**	0.42
		7	13.6 (2.6)	19.1 (1.2)	15.68	**<0.01**	0.49
Scrapes Created	Male	28	1.46 (0.12)	1.23 (0.15)	2.53	0.11	0.16
		7	1.54 (0.14)	1.26 (0.15)	2.23	0.14	0.18
	Female	28	1.25 (0.12)	1.34 (0.16)	0.01	0.95	0.01
		7	1.20 (0.14)	1.33 (0.16)	0.03	0.87	0.02
Duration of Visit	Male	28	58.2 (5.1)	48.0 (5.3)	1.44	0.23	0.12
		7	63.7 (6.2)	48.8 (5.2)	4.33	**0.04**	0.25
	Female	28	53.6 (5.0)	50.7 (5.8)	2.35	0.13	0.15
		7	56.4 (6.0)	50.4 (5.7)	1.92	0.17	0.17

Visitation behaviour variables include the days until next visit, the number of scrapes created, and duration of visit (in seconds). We tested the influence of whether females and other males had been present in two time periods: the previous month (28 days) and the previous week (7 days). We report the mean, standard error, p-value (significant values in bold), and effect size as Cohen’s d scores.

**Table 2:** The influences of visitation by male and female pumas on the display of four behaviours (scraping, body rubbing, investigating, and flehmen response) by male pumas.

Behavior	Sex	Days	%Displayed		*X*^*2*^	*p*	phi
			Present	Absent			
Scraping	Male	28	81.5%	76.1%	1.47	0.23	0.06
		7	81.5%	77.2%	0.49	0.49	0.04
	Female	28	77.1%	78.3%	0.03	0.86	0.01
		7	76.7%	78.2%	0.02	0.88	0.01
Body Rubbing	Male	28	15.9%	6.1%	10.52	**<0.01**	0.16
		7	12.3%	8.8%	0.61	0.43	0.05
	Female	28	6.9%	10.5%	1.11	0.29	0.06
		7	5.8%	10.2%	1.13	0.29	0.06
Investigating	Male	28	89.8%	88.7%	0.04	0.84	0.02
		7	91.4%	88.6%	0.28	0.60	0.03
	Female	28	90.3%	88.5%	0.16	0.69	0.03
		7	88.4%	89.2%	0.01	0.97	0.10
Flehmen Response	Male	28	10.8%	6.5%	2.17	0.14	0.08
		7	14.8%	6.5%	5.29	**0.02**	0.12
	Female	28	14.6%	5.0%	11.37	**<0.01**	0.17
		7	18.6%	5.5%	14.74	**<0.01**	0.19

We tested the influence of females and other males in two time periods, whether the other puma had been present in the previous month (28 days) and if they had been present in the previous week (7 days). For each behaviour we report the percent of visits where the behaviours were displayed, the p-value, and an effect size as *phi* coefficients.

**Table 3:** AIC models comparing the influences on the visitation and behaviours of male pumas.

Variable	Model	AIC	ΔAIC	AIC_*w*_
Days Until Next Visit	Male* Female* Season	141.44	10.05	0.01
	Male + Female + Season	137.95	6.56	0.04
	Male + Female	131.39	0.00	**0.95**
	Female* Season	341.63	210.24	0.00
	Female	319.76	188.37	0.00
	Male	358.10	226.71	0.00
Duration Of Visit	Male* Female* Season	88.76	27.32	0.00
	Male + Female + Season	71.13	9.69	0.01
	Male + Female	61.44	0.00	**0.99**
	Female* Season	160.88	99.44	0.00
	Female	136.70	75.26	0.00
	Male	198.53	137.09	0.00
Exhibiting Body Rubbing	Male* Female* Season	66.56	11.84	0.00
	Male + Female + Season	69.77	15.05	0.00
	Male + Female	54.72	0.00	**1.00**
	Female* Season	140.33	85.61	0.00
	Female	132.61	77.89	0.00
	Male	176.14	121.42	0.00
Exhibiting Flehmen Response	Male* Female* Season	50.23	5.28	0.06
	Male + Female + Season	49.46	4.51	0.09
	Male + Female	44.95	0.00	**0.85**
	Female* Season	119.76	74.81	0.00
	Female	122.74	77.79	0.00
	Male	165.88	120.93	0.00

Variables include the days until next visit (in days), and duration of visit (in seconds), and whether they displayed body rubbing, and the flehmen response. For each variable, we first tested five models to determine which was the best explanatory model, and then tested just male visitation versus female visitation. For each model we report the components of each model, the AIC score, the ΔAIC score, and the AIC weight (AIC_*w*_).
