# Supplementary material for: Evaluating the responses of a territorial solitary carnivore to potential mates and competitors
Source: Sci Rep. 2016 Jun 2;6:27257. doi: 10.1038/srep27257 (PMC4890113; doi:10.1038/srep27257)
Supplement: Supplementary Video Legends [file srep27257-s1.pdf]

Evaluating the responses of a territorial solitary carnivore to potential mates and competitors

Maximilian L. Allen, Veronica Yovovich, and Christopher C. Wilmers

Video legends

Video 1. Scraping, as shown in this video, is the most frequent form of scent marking used by pumas. Scraping is defined as when the puma claws the ground with their hind feet and then sometimes urinates and/or defecates on the scraped mound of material.

Video 2. Olfactory investigation, as shown in this video, is defined as when the puma uses its olfactory sense to investigate cues and signals; and is noted by the puma's nose within one head length of a scrape or other cue.

Video 3. This video shows a visit where a male puma exhibits the flehmen response, along with scraping and olfactory investigation. Flehmen response is defined as when the puma picks up its head and curls back its upper lip, in order to expose its vomeronasal organ and enhance its olfactory sense.

Video 4. Body rubbing, as shown in this video, is most frequently exhibited by male pumas, and is defined as when the puma rubbed its cheek or shoulder on the ground or an object, or rolled back and forth on the ground.
